# Assessing the mutagenic potential of methyl phenlactonoate 3 and Nijmegen-1 in bacterial reverse mutation assays

**DOI:** 10.1016/j.heliyon.2024.e40526

**Published:** 2024-11-20

**Authors:** Muhammad Jamil, Jian You Wang, Kinjal A. Patel, Rajendra M. Nagane, Manish V. Patel, Jalindar Totre, Satish E. Bhoge, Salim Al-Babili

**Affiliations:** a The BioActives Lab, BESE, King Abdullah University of Science and Technology, Thuwal, 23955-6900, Saudi Arabia; b Jai Research Foundation, Department of Toxicology, Valvada, 396 105, dist, Valsad, Gujarat, India; c UPL House, Express Highway, Bandra-East, Mumbai, 400 051, Maharashtra, India; d Plant Science Program, Biological and Environmental Science and Engineering Division, King Abdullah University of Science and Technology (KAUST), Thuwal, 23955-6900, Saudi Arabia

**Keywords:** Striga hermonthica, Strigolactone analogs, Methyl phenlactonoates, Nijmegen-1, Mutagenicity, Reverse mutation

## Abstract

The use of strigolactone (SL) analogs as suicidal germination agents to control the seed banks of the parasitic weed *Striga hermonthica* has gained interest for field applications in recent years. However, concerns about the environmental safety of these SL analogs remain. In this study, we evaluated the mutagenic potential of two selected SL analogs, Methyl Phenlactonoate 3 (MP3) and Nijmegen-1, across concentrations ranging from 1.5 to 5000 μg per plate. We conducted this assessment using five histidine-deficient mutant tester strains of *Salmonella typhimurium*. After incubating the SL analogs with the tester strains, we observed no significant increase in the number of revertants, with and without the S9 mix, compared to both negative and positive laboratory controls. These results suggest that MP3 and Nijmegen-1 are non-mutagenic according to the bacterial reverse mutation test, supporting their potential as environmentally safe agents for managing Striga populations.

## Highlights

• Strigolactones are important class of plant hormones.
• A lot of attention is being paid on understanding of their signalling, synthesis and biological roles.
• Understanding the mutagenic impact of strigolactone analogs is crucial for their general deployment in labs and fields.
• We investigated the mutagenic potential of two analogs using *Salmonella typhimurium* tester strains.
• The selected strigolactones are non-mutagenic to all tested strains of *Salmonella typhimurium* under the prescribed conditions.

## 1 Introduction

*Striga* spp. represent a group of root parasitic weeds from the *Orobanchaceae* family, prevalent throughout Africa, South and Eastern Europe, parts of Asia, and the Middle East [1,2]. These weeds afflict over 60 crop species, including staple cereals [3]. The seed germination process of *Striga hermonthica*, begins with a pre-conditioning phase in moist and warm conditions, allowing the seeds to respond to host-released germination stimulants, primarily strigolactones (SLs) [[4], [5], [6]]. As obligate parasites, germinated Striga must parasitize host roots to procure nutrients, failing which leads to their demise. Over the past decades, various SL analogs have been crafted to induce the lethal germination of these parasitic seeds, a method often termed suicidal germination [7,8]. This approach involves applying a synthetic germination stimulant to infested soil, triggering the germination of parasitic seeds, which subsequently perish due to the absence of a host [9]. Consequently, this strategy aims to diminish seed bank reserves and thereby reduce Striga infestations in affected fields [10].

SLs have been recognized as a new class of phytohormones, serving varied functions in plants and the rhizosphere [[11], [12], [13], [14], [15]], and are key germination stimulants for parasitic seeds. The focus on developing synthetic SL analogs has intensified recently [[16], [17], [18]]. Initial SL analogs, like GR24 and GR7, were either intricate or exhibited reduced activity [19]. Following the identification of carlactone as the core SL biosynthetic intermediate, a series of simple yet active carlactone-based germination stimulants, specifically methyl phenlactonoates (MPs), have been synthesized and evaluated against Striga [16,17,20]. Several potent MPs have since been formulated for field use [7]. A number of field trials aimed at assessing the potential suicidal germination effects of SL analogs to decrease Striga seed density in infested soils have been conducted in countries like Kenya, Burkina Faso, and Tanzania. Nonetheless, understanding the mutagenic impact of SL analogs is crucial for their commercial deployment in field settings. To our knowledge, the mutagenic potential of SL analogs has not been explored, necessitating extensive research in this area to ensure their safe application as suicidal agents in the future.

The bacterial reverse mutation test, widely used to detect point mutations in amino acid-dependent strains of *Salmonella typhimurium* and *Escherichia coli*, is well-known for its reliability and reproducibility in short-term mutagenicity assessments. It is endorsed by the Organisation for Economic Co-operation and Development (OECD) and other regulatory bodies [[21], [22], [23]]. This test is commonly applied in primary screenings for genotoxic activity, particularly to identify substances that induce point mutations [24,25]. The test works on the principle that mutations in the tester strains can revert, restoring the bacteria's ability to synthesize essential amino acids [26]. We identify revertant bacteria by their ability to grow in the absence of the amino acids required by the parent tester strains. In this study, we evaluated the mutagenic potential of Methyl Phenlactonoate 3 (MP3) and Nijmegen-1 using *Salmonella typhimurium* tester strains TA1537, TA1535, TA98, TA100, and TA102, both with and without a metabolic activation system (Supplementary Tables 1–2). Our goal was to assess the ability of MP3 and Nijmegen-1 to induce reverse mutations at specific histidine loci, determining their safety and efficacy.

## 2 Materials and methods

### 2.1 Strigolactone analogs synthesis process and structural characterization

The two strigolactone (SL) analogs, Methyl Phenlactonoate 3 (MP3) and Nijmegen-1, were chosen and synthesized at UPL-India. The standard procedure for synthesis of MP3 and Nijmegen-1 is shown below.

A) Standard procedure for synthesis of MP3

Step-1**& insitu**Step-2: Preparation of Methyl 3-Hydroxy-3-phenylprop-2-enote.

**Figure:**
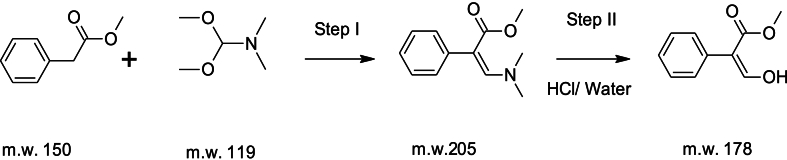

Step-3: Preparation of MP-3: Benzene acetic acid, α-[[(2,5-dihydro-4-methyl-5-oxo-2-furanyl) oxy] methyl-ene] methyl ester.

**Figure:**
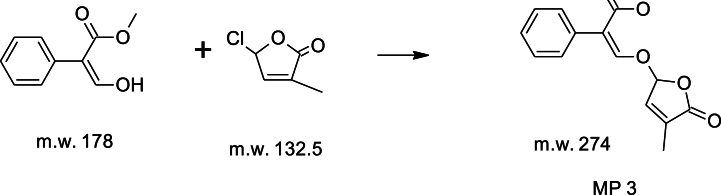

We added anhydrous potassium carbonate (135 g, 0.95 mol) to a stirred solution of Sheehan aldehyde (120 g, 0.60 mol) and EDTA (0.80 g) in toluene (750 ml) at room temperature under nitrogen. We then gradually added chlorofuranone (97 g, 0.71 mol) over 2 h while maintaining the temperature between 80 and 85 °C. We stirred the mixture at this temperature for 6–8 h. After cooling the reaction mass to 35–40 °C, we diluted it with ethyl acetate (330 ml) and water (300 ml). We separated the organic layer, extracted the aqueous layer with ethyl acetate (330 ml), and combined the organic phases for concentration under reduced pressure. We dissolved the crude mass in 2-propanol (240 ml) at 85–90 °C and allowed it to crystallize by cooling to 0–5 °C. We filtered the precipitate, washed it with 2-propanol (40 ml), and dried it to obtain 100 g of MP3 as a white solid.

mp 88–90 °C; ^1^H NMR (CDCl_3_, 500 MHz) δ 1.97 (br s, 3H, CH_3_), 3.76 (s, 3H, OCH_3_), 6.13 (s, 1H, OCHO), 6.85 (s, 1H, =CH), 7.30 (m, 3H, 2 Aromatic H), 7.34 (m, 2H, 2 Aromatic H), 7.74 (s, 1H, =CHO); MS [EI, *m*/*z*] 274 ([M]+).

B) Standard Procedure for Synthesis of Nijmegen-1

The synthesis of Nijmegen-1 (Technical) starting Phthalimide is conducted in three distinct synthesis steps as described below.

Step 1 Preparation of Methyl 2-(1,3-dioxoisoindolin-2-yl) acetate

**Figure:**
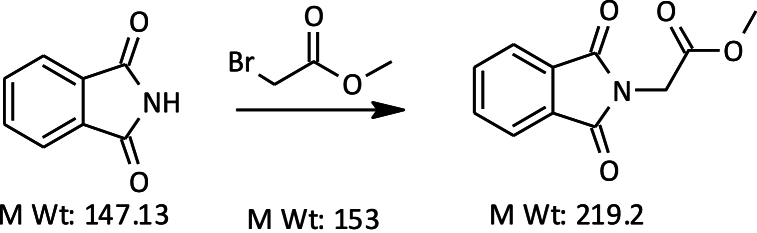

Step-2 Preparation of Methyl (E)-2-(1,3-dioxoisoindolin-2-yl)-3-hydroxy-prop-2-enoate (Sheehan aldehyde).

**Figure:**
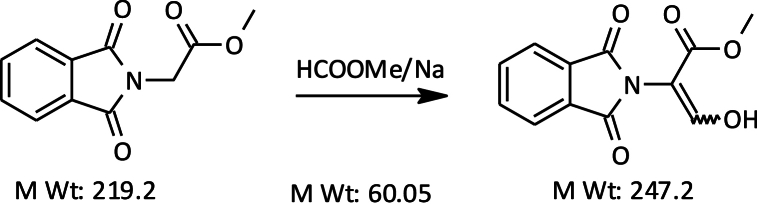

Step 3 Preparation of Nijmegen-1

**Figure:**
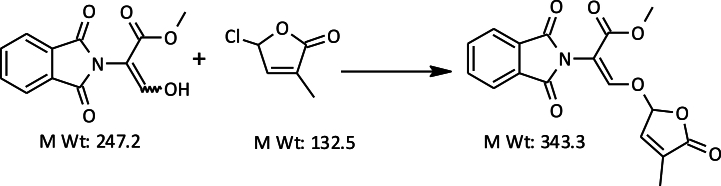

#### 2.1.1 Preparation of methyl 2-(1,3-dioxoisoindolin-2-yl) acetate

We added powdered potassium carbonate (120 g, 0.85 mol) and EDTA (0.2 g) to a solution of phthalimide (116 g, 0.77 mol) in DMF (490 ml). We then heated the reaction mixture to 50 °C and added methyl bromoacetate (141 g, 0.89 mol) dropwise over 20–30 min. We continued heating the reaction mixture to 90 °C for 6 h until the reaction was complete. After cooling, we diluted the mixture with water (600 g) to precipitate the product. We filtered the precipitate, washed it with water (100 g), and dried it under vacuum to obtain 150 g of methyl 2-(1,3-dioxoisoindolin-2-yl) acetate as an off-white solid, with an 85 % isolated yield and 96 % purity by a/a.

#### 2.1.2 Preparation of methyl 2-(1,3-Dioxo-1,3-dihydroisoindol-2-yl)-3-oxopropionate (Sheehan aldehyde)

We prepared a cooled (0 °C) solution of methyl 2-(1,3-dioxoisoindolin-2-yl) acetate (150 g, 0.65 mol) in methyl formate (390 ml). To this solution, we slowly added EDTA (0.25 g) and small pieces of sodium (23 g, 0.99 mol), with mechanical stirring under a nitrogen atmosphere. We continued stirring for 18 h until all the sodium had dissolved. Next, we concentrated the reaction mixture under reduced pressure, and treated the resulting residue with dichloromethane (330 ml) and a mixture of glacial acetic acid (150 ml) and 30 % HCl (49 ml). We isolated the crude Sheehan aldehyde by extracting with dichloromethane (2 × 200 ml), then dried the organic phase over Na₂SO₄ and concentrated it under vacuum. Finally, we recrystallized the product from toluene (220 ml) to obtain pure Sheehan aldehyde (119 g, 70 %) as a pale-yellow powder, with physical properties matching those previously reported [27].

#### 2.1.3 Preparation of methyl 2-(1,3-Dioxo-1,3-dihydroisoindol-2-yl)-3-[4-methyl-5-oxo-2,5-dihydrofuran-2(R)-yloxy] acrylate (Nijmegen-1)

We added anhydrous potassium carbonate (100 g, 0.71 mol) to a stirred solution of Sheehan aldehyde (119 g, 0.45 mol) and EDTA (0.45 g) in toluene (650 ml) at room temperature under nitrogen. We then gradually added chlorofuranone (72 g, 0.52 mol) in toluene (80 ml) over 2 h, maintaining the temperature between 80 and 85 °C. We stirred the mixture at this temperature for 6–8 h. Afterward, we cooled the mixture to 35–40 °C, diluted it with ethyl acetate (550 ml) and water (500 ml), separated the organic phase, and concentrated it under reduced pressure. We dissolved the resulting oily residue in 2-propanol (650 ml) at 85–90 °C and allowed it to crystallize as it cooled to 0–5 °C. Finally, we filtered the precipitate, washed it with 2-propanol (60 ml), and dried it to obtain Nijmegen-1 as a white solid.

mp 151–152 °C; ^1^H NMR (CDCl_3_, 500 MHz) δ 1.97 (br s, 3H, CH_3_), 3.79 (s, 3H, OCH_3_), 6.17 (br s, 1H, OCHO), 6.90 (br s, 1H, =CH), 7.77 (m, 2H, 2 Aromatic H), 7.90 (m, 3H, 2 Aromatic H + 1H =CHO); MS [EI, *m*/*z*] 343 ([M]+).

### 2.2 Structural characterization of SL analogs

The molecular formula of the two synthesized compounds (MP3 and Nijmegen-1) is confirmed by characterization using NMR and mass spectroscopy and confirmed with the data published before [16,18,28].

^1^H NMR (CDCl_3_, 400 MHz) δ 1.97 (br s, 3H, CH_3_), 3.78 (s, 3H, OCH_3_), 6.17 (br s, ^1^H, OCHO), 6.90 (br s, 1H, =CH), 7.76 (m, 2H, 2 arom H), 7.90 (m, 3H, 2 arom H + =CHO); Anal. Calcd for C_17_H_13_NO_7_: C, 59.48; H, 3.82; N, 4.08. Found: C, 59.10; H, 3.85; N, 4.00.

The structural characterization of the two compounds by ^1^H NMR is indicated in Table 1 and Supplementary Figs. 1 and 2. The additional information on safety of both MP3 and Nijmegen-1 are presented as safety data sheet in Supplementary files S1 and S2.

**Table 1:** Characterization of MP-3 and Nijmegen-1 by ^1^H NMR. Details of chemical shifts for ^1^H NMR spectra.

Sr.	Name	Structure	Chemical Shift (ppm)	Protons	Description
**1.**	**MP3** **IUPAC Name:** Methyl 3-(4-methyl-5-oxo-2,5-dihydrofuran-2-yloxy)-2-phenylacrylate **Mol. Formula:** C_15_H_14_O_5_		1.959	3	One methyl group protons attached to the furan ring
			3.755–3.757	3	One methyl group protons from methyl ester group
			6.123	1	One -CH group proton from furan ring attached to oxygen atom
			6.840–6.844	1	One -CH group proton attached to oxygen atom.
			7.257–7.346	5	Five aromatic protons from benzene ring
			7.741–7.743	1	One -CH group proton from furan ring
**2.**	**Nijmegen-1** **IUPAC Name:** 2H-Isoindole-2-acetic acid, α-[[(2,5-dihydro-4-methyl-5-oxo-2-furanyl) oxy] methylene]-1,3-dihydro-1,3-dioxo-, methyl ester **Mol. Formula:** C_17_H_13_NO_7_		1.960–1.963	3	One methyl group protons attached to the furan ring
			3.768–3.773	3	One methyl group protons from methyl ester group
			6.180–6.183	1	One -CH group proton from furan ring attached to oxygen atom
			6.900	1	One -CH group proton attached to oxygen atom.
			7.748–7.908	5	One -CH group proton from furan ring and four aromatic protons from benzene ring

### 2.3 Study sites and bacterial strains

The Department of Toxicology at Jai Research Foundation, Valvada, 396,105 Dist. Valsad, Gujarat, India, conducted this study. It followed the OECD 471, 2020 guidelines, specifically the Bacterial Reverse Mutation Test as outlined by the Organization for Economic Co-operation and Development (OECD), adopted on July 21, 1997, and amended on June 26, 2020 (CAS RN Paragraph 24). The strains used in this study were obtained from Molecular Toxicology, Inc. (157 Industrial Park Dr., Boone, North Carolina, NC 28607, U.S.A.). The *Salmonella typhimurium* mutant strains employed were derived from the *Salmonella typhimurium* strain LT2. These tested mutant strains required histidine supplementation for growth. Each assay included both positive (strain-specific) and negative (vehicle) controls, with and without metabolic activation. The concurrent negative control, which contained only the vehicle and no test item, was treated identically to the treatment groups in each assay. (Supplementary Table 3).

### 2.4 Metabolic activation system

Unlike mammals, bacteria lack the oxidative enzyme systems needed to metabolize exogenous compounds into electrophilic metabolites that can interact with DNA. In mammals, however, these compounds can sometimes undergo metabolic activation by enzyme systems to form mutagenic products. To replicate this mammalian metabolic process in bacterial assays, researchers add the S9 fraction, which is buffered and enriched with essential cofactors like β-NADP and glucose-6-phosphate, collectively known as the "S9 mix" (Supplementary Table 4). This mix is then incorporated into top agar (Supplementary Table 5) for the activation assays. In this study, we sourced the S9 fractions from Meshram Genotox Services (Nagpur, Maharashtra) (Lot N° MWR/ARI/S9F/01/19), as detailed in Supplementary Table 6.

### 2.5 Solubility and precipitation test

Both the SL analogs MP3 and Nijmegen-1 showed insolubility in distilled water (refer to as Stock A, 50,000 μg/ml) but dissolved in dimethyl sulfoxide (DMSO) (refer to as Stock B, 50,000 μg/ml) [29]. As a result, we selected DMSO for further treatments. To assess precipitation, we added 100 μl from Stock B to 2 ml of top agar and applied the mixture to a Minimal Glucose agar plate. No precipitation occurred at the tested concentration of 5000 μg/plate. Therefore, we determined 5000 μg/plate as the maximal concentration for mutagenicity testing, both with and without metabolic activation [29].

### 2.6 Cell viability test

We initiated fresh cultures by inoculating frozen permanent cultures into flasks containing 10 ml of sterile Nutrient Broth No. 2 (Oxoid). We then incubated these flasks at 37 ± 1 °C in an orbital shaking incubator set to 120 rpm for 15 h, allowing the cultures to reach either early stationary or late exponential phase. After incubation, we removed the culture flasks from the incubator. We diluted the cultures with Oxoid Nutrient Broth (ONB) and measured the optical density at 660 nm using a UV/visible spectrophotometer (V-650 Series, Jasco), with Oxoid Nutrient Broth serving as the control blank. We confirmed the viability of the test strains before treatment. Since the optical density of the cultures fell within the acceptable range, we deemed them suitable for the study (Supplementary Fig. 3).

### 2.7 Genotype confirmation test

We routinely verified the genotypes of all tester strains on a monthly basis [29]. We assessed the *Salmonella typhimurium* tester strains for various genetic traits, including biotin dependence, histidine dependence, the presence of the rfa mutation, dual dependence on histidine and biotin, the uvrB, and the R-factor that confers resistance to antibiotics such as ampicillin and tetracycline [29,30]. We followed the Jai Research Foundation standard operating procedure (JRF/MIC/SOP-611) for genotype confirmation. The results of the most recent genotype confirmations are also documented (Table 2).

**Table 2:** Testing confirmation of genotype for tester strains (MP3 and Nijmegen-1).

Name of Test	*Salmonella typhimurium* tester strains				
	TA1537	TA1535	TA98	TA100	TA102
Histidine Dependence	NG	NG	NG	NG	NG
Biotin Dependence	NG	NG	NG	NG	G
Histidine and Biotin Dependence	G	G	G	G	G
*rfa* Mutation	ZI	ZI	ZI	ZI	ZI
DNA repair (*uvrB*)	NG∗	NG∗	NG∗	NG∗	G
R – factor Resistance					
Ampicillin Resistance	NG	NG	G	G	G
Tetracycline Resistance	NG	NG	NG	NG	G

NG: No Growth; G: Growth; ZI: Zone of Inhibition; ∗: On Irradiated Side.

(Results: Strains have retained their genetic characteristics).

### 2.8 Mutagenicity test

We assessed the mutagenicity of SL analogs using the plate incorporation method with all five tester strains of *Salmonella typhimurium*. We performed the experiment both with and without a metabolic activation system (5 % v/v S9 mix). To prepare the primary stock solution (Stock A), we dissolved 500 mg of the test item in dimethyl sulfoxide (DMSO) and adjusted the volume to 10 ml, resulting in a concentration of 50,000 μg/ml. We made further dilutions for stock solutions B–H (Supplementary Table 7). We kept tubes containing 2 ml of molten top agar with 0.5 mM histidine/biotin at 45 ± 2 °C. For conditions without metabolic activation, we added 500 μl of 0.2 M phosphate buffer, and for conditions with metabolic activation, we incorporated 500 μl of 5 % v/v S9 mix. We treated the samples with 100 μl from the relevant stock solution of the test item, DMSO (as a negative control), and the appropriate positive control. We then added 100 μl of bacterial culture to each tube and mixed thoroughly. The treatment mixture was poured onto Minimal Glucose Agar (MGA) plates and allowed to solidify. We prepared duplicate sets of plates for each test item, positive control, and negative control. We incubated the Petri plates at 37 ± 1 °C for 48 h, then examined them to evaluate any background bacterial lawn inhibition and reduction in colony count.

### 2.9 Assay evaluation and acceptance criteria

We considered a result positive if there was a concentration-dependent increase throughout the tested range and/or a consistent elevation in the number of revertant colonies per plate at one or more concentrations in at least one strain, with or without a metabolic activation system [29]. For strains TA1535 and TA1537, we classified datasets as positive if the peak increase in mean revertants in the dose-response was equal to or greater than 3.0 times the mean value of the negative control. For strains TA98, TA100, and TA102, we classified datasets as positive if the peak increase in mean revertants was at least 2.0 times the mean negative control value.

Before assessing the assay data, we ensured all criteria for a valid assay were met. All *Salmonella typhimurium* tester strain cultures showed sensitivity to crystal violet, confirming the presence of the rfa wall mutation (Table 1). All tester strains required biotin, except for strain TA102, which was biotin-independent. The tester strains exhibited sensitivity to UV exposure, except for the wild-type strain TA102. All tester strains required histidine for growth. Strains TA98, TA100, and TA102 were resistant to ampicillin, indicating the presence of the pKM101 plasmid. TA102 also showed resistance to tetracycline, signifying the presence of the pAQ1 plasmid. All negative control cultures showed a typical number of spontaneous revertants per plate, confirming the histidine requirement of *Salmonella typhimurium* (Supplementary Table 3).

The optical densities (OD at 660 nm) of all tester strains fell within the specified range, ensuring cultures contained approximately 1–2 × 10⁹ bacterial cells/ml and that we plated an appropriate number of bacteria (Supplementary Fig. 3). The mean value of the positive control for each tester strain showed a significant increase compared to the negative control, demonstrating the tester strains' ability to detect mutagens. Additionally, the mean value of the positive control for each strain was markedly higher than the negative control, confirming that the S9 mix effectively metabolized a pro-mutagen into its mutagenic forms. We also evaluated a suitable positive control for each strain in the presence of S9 to verify the integrity of the S9 mix and the tester strain's ability to identify mutagens.

### 2.10 Cytotoxicity evaluation criteria

We assessed the assay data using six analyzable doses [29]. We characterized cytotoxicity by either a reduction of more than 50 % in the average number of revertants per concentration compared to the mean value of the negative control or by a decreased density of the bacterial background lawn [31].

### 2.11 Statistical analysis

Statistical analysis was employed to assess the dose–response relationship. Only responses meeting all three specified criteria magnitude, concentration responsiveness, and reproducibility were considered positive for evaluation.

## 3 Results and discussion

### 3.1 Bacterial cell viability test

We ensured the cell densities (OD at 660 nm) of all tester strains fell within the required range to yield cultures with approximately 1–2 × 10⁹ bacterial cells/ml, confirming that we plated an adequate number of bacteria (Supplementary Fig. 3). We confirmed the viability of the test strains prior to treatment. Since the optical density of the cultures was within the acceptable range, we deemed them suitable for the study (Supplementary Fig. 3). We used cultivation-based and optical assays [32], which are commonly employed methods for evaluating bacterial viability [33]. These bacterial cell viability assays are primarily used to assess the overall metabolic activity of microorganisms [34]. These results confirmed the optimal bacterial cell density required to generate reliable data on the application of SLs.

### 3.2 Genotype confirmation test

*We widely use Salmonella typhimurium strains in the Ames assay, a test that evaluates mutagenicity based on histidine synthesis* [23]. Each tester strain can detect mutations in histidine, as well as other genetic alterations. All *Salmonella typhimurium* tester strain cultures showed sensitivity to crystal violet, confirming the presence of RFA wall mutations. All tester strains, except for TA102, depended on biotin. We observed sensitivity to UV exposure in all tester strains, except for the wild-type strain TA102. All tester strains required histidine for growth. Strains TA98, TA100, and TA102 exhibited resistance to ampicillin, confirming the presence of the pKM101 plasmid. Additionally, TA102 showed resistance to tetracycline, indicating the presence of the pAQ1 plasmid. We incorporated the results from the most recent genotype testing into this study (Table 1).

### 3.3 Bacterial mutagenicity

We assessed an adequate positive control for each strain both with and without the S9 mix, validating the integrity of the S9 mix and the strain's ability to detect mutagens. The mean value of the positive control, both with and without a metabolic activation system (±S9), for each tester strain showed a significant increase above the corresponding strain's negative control. This confirmed that the tester strains were capable of identifying mutagens and that the S9 mix effectively converted a pro-mutagen into its mutagenic form(s). The study's findings further confirmed that the negative control values for all strains fell within the historical range for the respective strains (Supplementary Table 3).

**Fig. 1:**
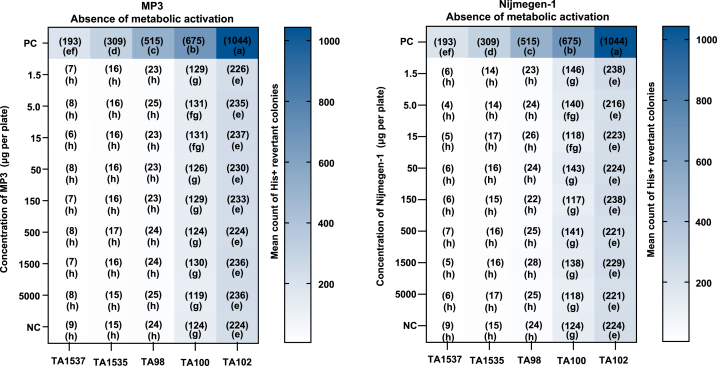
Mean count of His + revertant colonies per plate in response to various concentrations of MP3 and Nijmegen-1 (absence of metabolic activation). Plates containing different strains were exposed to varying concentrations of the two SL analogs. Values in parentheses in each box denote the mean count of His + revertant colonies per plate (n = 2). For each strigolactone analog, treatments with distinct letters indicate significant differences (p < 0.05). Dimethyl sulfoxide served as the negative control (NC). Positive Controls (PC): TA1537 = 9-Aminoacridine Hydrochloride Monohydrate (75 μg/plate), TA1535 = Sodium Azide (0.5 μg/plate), TA98 = 2-Nitrofluorene (7.5 μg/plate), TA100 = Sodium Azide (5 μg/plate), TA102 = Mitomycin-C (0.5 μg/plate), 2-Aa = 2-Aminoanthracene (5 μg/plate for TA100).

We calculated the mean number of histidine-revertant colonies per plate at various concentrations during the mutagenicity test. No significant impact of SLs on any of the tester strains was observed. The results showed no increase in revertant colonies (i.e., no mutagenic effect) with or without the metabolic activation system (5 % v/v S9 mix) in any tester strain. Similarly, no positive mutagenic effect was detected in any tester strain, even at the highest tested concentration of 5000 μg per plate of MP3 and Nijmegen-1, with or without the metabolic activation system, when compared to the negative control. The mean number of histidine-revertant colonies at different test concentrations is shown in Fig. 1. The regression equation for revertants (y) in relation to concentration (x) is provided in Supplementary Table 8.

**Fig. 2:**
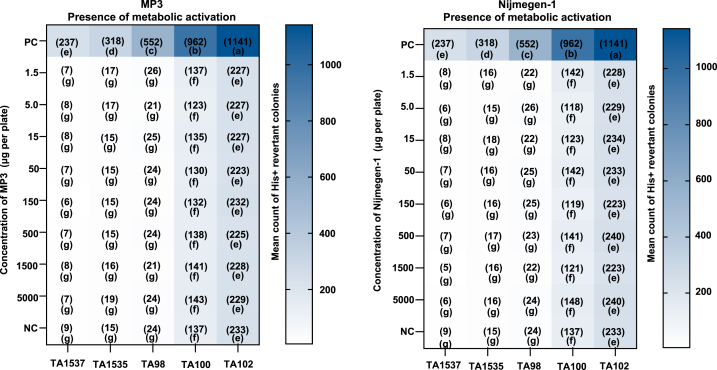
Mean count of His + revertant colonies per plate in response to various concentrations of MP3 and Nijmegen-1 (presence of metabolic activation). Plates containing different strains were treated with varying concentrations of the two SL analogs. Values in parentheses in each box represent the mean count of His + revertant colonies per plate (n = 2). For each strigolactone analog, treatments with distinct letters show significant differences (p < 0.05). Dimethyl sulfoxide served as the negative control (NC). Positive Control (PC): 2-Aa = 2-Aminoanthracene (10 μg/plate for TA1537, TA1535, TA102, and 5 μg/plate for TA98 and TA100).

In the mutagenicity test, we used 2-aminoanthracene as a positive control with the metabolic activation system for the tester strains. The positive controls showed a significant increase in revertant numbers compared to the negative controls, confirming the efficacy of the test system and the appropriateness of the methods used. In the TA100 cells (mutagenicity test), no significant increase in revertants was observed when treated with 2-aminoanthracene without metabolic activation. However, with metabolic activation, we observed a substantial increase in revertants, underscoring the effectiveness of the S9 fraction used in this assay. The mean count of histidine-revertant colonies in the positive controls for the mutagenicity test is shown in Fig. 2.

Bacterial mutagenicity has played a central role in numerous large-scale trials in recent years [35,36]. Researchers have added features to enhance bacterial sensitivity to mutations induced by chemical agents. For example, many carcinogens (or their metabolites) are large molecules that struggle to penetrate bacterial cell walls [37]. Wild-type cells produce lipopolysaccharides that act as barriers to bulky hydrophobic molecules. To address this, we introduced the rfa mutation in *Salmonella* strains, resulting in defective lipopolysaccharides and increased permeability. Since SL analogs are generally small molecules, the proposed bacterial tests are well-suited to yield reliable data on bacterial mutagenicity.

### 3.4 Bacterial background, lawn pattern, and percent reduction

**Fig. 3:**
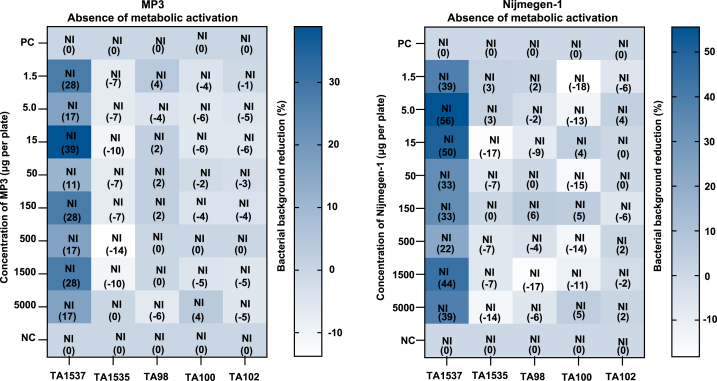
Influence of MP3 and Nijmegen-1 on the bacterial background lawn pattern and percent reduction without metabolic activation system. Dimethyl sulfoxide served as the negative control (NC). Percent reductions are indicated in parentheses (n = 2), NI = No Inhibition. Positive Controls (PC): TA1537 = 9-Aminoacridine Hydrochloride Monohydrate (75 μg/plate), TA1535 = Sodium Azide (0.5 μg/plate), TA98 = 2-Nitrofluorene (7.5 μg/plate), TA100 = Sodium Azide (5 μg/plate), TA102 = Mitomycin-C (0.5 μg/plate), 2-Aa = 2-Aminoanthracene (5 μg/plate for TA100).

Cytotoxicity was indicated by inhibition of the bacterial background, changes in the lawn pattern, and/or a reduction in the number of revertant colonies. The percentage decrease in revertant colonies and the lawn patterns for all *Salmonella typhimurium* tester strains, both with and without the metabolic activation system (5 % v/v S9 mix), are shown in Fig. 3.

**Fig. 4:**
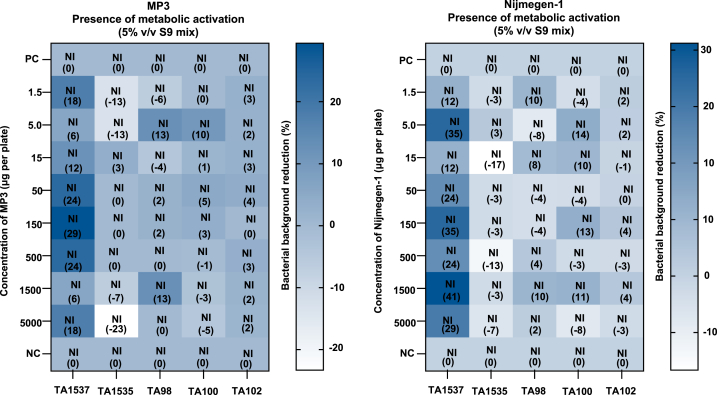
Impact of MP3 and Nijmegen-1 on the bacterial background lawn pattern and percent reduction in the presence of a metabolic activation system (5 % v/v S9 mix). Dimethyl sulfoxide was included as the negative control (NC). Percent reductions are specified in parentheses (n = 2), NI = No Inhibition. Positive Control (PC): 2-Aa = 2-Aminoanthracene (10 μg/plate for TA1537, TA1535, TA102, and 5 μg/plate for TA98 and TA100).

The data showed no positive mutagenic effect in any tester strain, even at the highest tested concentration of 5000 μg/plate for MP3 and Nijmegen-1, regardless of the presence or absence of the metabolic activation (5 % v/v S9 mix), when compared to the negative control. Statistical analyses revealed no significant effects in tester strains TA1537, TA1535, TA100, and TA102 without metabolic activation, nor in tester strains TA1537, TA98, TA100, and TA102 with metabolic activation. In tester strains TA98 (without metabolic activation) and TA1535 (with metabolic activation), the statistical analysis showed a 5 % correlation. However, the mean values were consistent with historical control data, making this finding biologically insignificant (Fig. 4).

DNA replication plays a key role in mutagenesis, while the background lawn is important for indicating growth inhibition due to the tested chemicals. An increase in histidine on the plate could elevate mutagenicity, but it may also cause significant growth of the background lawn, potentially obscuring the detection of revertants. These findings align with previously established procedures [22].

## 4 Conclusions

In summary, the SL analogs, MP3 and Nijmegen-1, did not cause a significant increase in the number of revertants in any tester strain, regardless of the presence or absence of the S9 mix. As a result, our findings show that MP3 and Nijmegen-1 are non-mutagenic to all tested *Salmonella typhimurium* strains—TA1537, TA1535, TA98, TA100, and TA102—under the prescribed conditions. Therefore, these SL analogs can be considered environmentally safe agents for managing Striga in infested fields.

## CRediT authorship contribution statement

**Muhammad Jamil:** Writing – review & editing, Writing – original draft, Validation, Resources, Methodology, Investigation. **Jian You Wang:** Writing – original draft, Validation, Data curation. **Kinjal A. Patel:** Methodology, Investigation, Formal analysis, Data curation. **Rajendra M. Nagane:** Methodology, Investigation, Formal analysis, Data curation. **Manish V. Patel:** Methodology, Investigation, Formal analysis, Data curation. **Jalindar Totre:** Validation, Resources, Methodology. **Satish E. Bhoge:** Resources, Investigation, Formal analysis. **Salim Al-Babili:** Writing – review & editing, Writing – original draft, Supervision, Resources, Project administration, Funding acquisition, Conceptualization.

## Data availability statement

The data will be available upon request. All data generated or analyzed during this study are included in the published article.

## Funding

The 10.13039/100000865Bill & Melinda Gates Foundation (grant number OPP1136424) and baseline funding from 10.13039/501100004052King Abdullah University of Science and Technology provided financial support for this research to S. A.-B.

## Declaration of competing interest

The authors declare the following financial interests/personal relationships which may be considered as potential competing interests:Salim Al-Babili reports financial support was provided by 10.13039/100000865Bill and Melinda Gates Foundation. Salim Al-Babili has patent #EP17784016.2/3519396 issued to KING ABDULLAH UNIVERSITY OF SCIENCE AND TECHNOLOGY UNIVERSITY OF TOKYO. If there are other authors, they declare that they have no known competing financial interests or personal relationships that could have appeared to influence the work reported in this paper.

## Abbreviations

SL: strigolactone
MP: Methyl Phenlactonoate
OECD: Organisation for Economic Co-operation and Development
DMSO: dimethyl sulfoxide
DNA: Deoxyribonucleic acid
NADP: Nicotinamide adenine dinucleotide phosphate
MGA: Minimal Glucose Agar
OD: optical density
UV: Ultraviolet
ml: milliliter
μl: microliter
M: Molar
mM: millimolar
μg: microgram
mg: milligram
v/v: vol/vol
h: hour
ONB: Oxoid Nutrient Broth

## References

[1] Teka H.B. (2014). Advance research on Striga control: a review. Afr. J. Plant Sci..

[2] Ejeta G. (2007). The Striga scourge in Africa: a growing pandemic, Integrating new technologies for Striga control: towards ending the witch-hunt. World Scientific.

[3] Rubiales D., Fernández-Aparicio M., Vurro M., Eizenberg H. (2018). Advances in parasitic weed research. Front. Plant Sci..

[4] Brun G., Braem L., Thoiron S., Gevaert K., Goormachtig S., Delavault P. (2018). Seed germination in parasitic plants: what insights can we expect from strigolactone research?. J. Exp. Bot..

[5] Kgosi R.L., Zwanenburg B., Mwakaboko A.S., Murdoch A.J. (2012). Strigolactone analogues induce suicidal seed germination of Striga spp. soil, Weed Res.

[6] Matusova R., van Mourik T., Bouwmeester H.J. (2004). Changes in the sensitivity of parasitic weed seeds to germination stimulants. Seed Sci. Res..

[7] Kountche B.A., Jamil M., Yonli D., Nikiema M.P., Blanco‐Ania D., Asami T., Zwanenburg B., Al‐Babili S. (2019). Suicidal germination as a control strategy for Striga hermonthica (Benth.) in smallholder farms of sub‐Saharan Africa. Plants, People, Planet.

[8] Zwanenburg B., Mwakaboko A.S., Kannan C. (2016). Suicidal germination for parasitic weed control. Pest Manag. Sci..

[9] Jamil M., Wang J.Y., Yonli D., Ota T., Berqdar L., Traore H., Margueritte O., Zwanenburg B., Asami T., Al-Babili S. (2022). Striga hermonthica suicidal germination activity of potent strigolactone analogs: evaluation from laboratory bioassays to field trials. Plants.

[10] Jamil M., Wang J.Y., Yonli D., Patil R.H., Riyazaddin M., Gangashetty P., Berqdar L., Chen G.-T.E., Traore H., Margueritte O. (2022). A new formulation for strigolactone suicidal germination agents, towards successful Striga management. Plants.

[11] Xie X.N., Yoneyama K., Yoneyama K., VanAlfen N.K., Bruening G., Leach J.E. (2010). The Strigolactone Story.

[12] Al-Babili S., Bouwmeester H.J. (2015). Strigolactones, a novel carotenoid-derived plant hormone. Annu. Rev. Plant Biol..

[13] Wang J.Y., Lin P.-Y., Al-Babili S. (2021). On the biosynthesis and evolution of apocarotenoid plant growth regulators. Semin. Cell Dev. Biol..

[14] Ito S., Braguy J., Wang J.Y., Yoda A., Fiorilli V., Takahashi I., Jamil M., Felemban A., Miyazaki S., Mazzarella T. (2022). Canonical strigolactones are not the major determinant of tillering but important rhizospheric signals in rice. Sci. Adv..

[15] Chen G.-T.E., Wang J.Y., Votta C., Braguy J., Jamil M., Kirschner G.K., Fiorilli V., Berqdar L., Balakrishna A., Blilou I. (2023).

[16] Jamil M., Kountche B.A., Haider I., Guo X.J., Ntui V.O., Jia K.P., Ali S., Hameed U.S., Nakamura H., Lyu Y., Jiang K., Hirabayashi K., Tanokura M., Arold S.T., Asami T., Al-Babili S. (2018). Methyl phenlactonoates are efficient strigolactone analogs with simple structure. J. Exp. Bot..

[17] Jamil M., Kountche B.A., Wang J.Y., Haider I., Jia K.-P., Takahashi I., Ota T., Asami T., Al-Babili S. (2020). A new series of carlactonoic acid based strigolactone analogs for fundamental and applied research. Front. Plant Sci..

[18] Zwanenburg B., Mwakaboko A.S. (2011). Strigolactone analogues and mimics derived from phthalimide, saccharine, p-tolylmalondialdehyde, benzoic and salicylic acid as scaffolds. Bioorg. Med. Chem..

[19] Zwanenburg B., Pospisil T., Zeljkovic S.C. (2016). Strigolactones: new plant hormones in action. Planta.

[20] Alder A., Jamil M., Marzorati M., Bruno M., Vermathen M., Bigler P., Ghisla S., Bouwmeester H., Beyer P., Al-Babili S. (2012). The path from β-carotene to carlactone, a strigolactone-like plant hormone. Science.

[21] Ames B.N., McCann J., Yamasaki E. (1975). Methods for detecting carcinogens and mutagens with the Salmonella/mammalian-microsome mutagenicity test. Mutat. Res..

[22] Maron D.M., Ames B.N. (1983).

[23] Vijay U., Gupta S., Mathur P., Suravajhala P., Bhatnagar P. (2018). Microbial mutagenicity assay: Ames test. Bio-protocol.

[24] Gatehouse D., Haworth S., Cebula T., Gocke E., Kier L., Matsushima T., Melcion C., Nohmi T., Ohta T., Venitt S. (1994). Recommendations for the performance of bacterial mutation assays. Mutat. Res. Environ. Mutagen Relat. Subj..

[25] Kato M., Sugiyama K.-i., Fukushima T., Miura Y., Awogi T., Hikosaka S., Kawakami K., Nakajima M., Nakamura M., Sui H. (2018). Negative and positive control ranges in the bacterial reverse mutation test: JEMS/BMS collaborative study. Gene Environ..

[26] Hamel A., Roy M., Proudlock R. (2016). Genetic Toxicology Testing.

[27] Sheehan J.C., Johnson D.A. (1954). The synthesis of substituted penicillins and simpler structural analogs. VIII. Phthalimidomalonaldehydic esters: Synthesis and condensation with penicillamine, Journal of the American Chemical Society.

[28] Nefkens G.H., Thuring J.W.J., Beenakkers M.F., Zwanenburg B. (1997). Synthesis of a phthaloylglycine-derived strigol analogue and its germination stimulatory activity toward seeds of the parasitic weeds Striga hermonthica and Orobanche crenata. J. Agric. Food Chem..

[29] Agency E.C. (2020).

[30] Mortelmans K., Zeiger E. (2000). The Ames Salmonella/microsome mutagenicity assay. Mutation research/fundamental and molecular mechanisms of mutagenesis.

[31] Teo S.K., San R.H., Wagner V.O., Gudi R., Stirling D.I., Thomas S.D., Khetani V.D. (2003). D-Methylphenidate is non-genotoxic in in vitro and in vivo assays. Mutat. Res. Genet. Toxicol. Environ. Mutagen.

[32] Müller S., Hübschmann T., Kleinsteuber S., Vogt C. (2012). High resolution single cell analytics to follow microbial community dynamics in anaerobic ecosystems. Methods.

[33] Qiu T.A., Nguyen T.H.T., Hudson-Smith N.V., Clement P.L., Forester D.-C., Frew H., Hang M.N., Murphy C.J., Hamers R.J., Feng Z.V. (2017). Growth-based bacterial viability assay for interference-free and high-throughput toxicity screening of nanomaterials. Anal. Chem..

[34] Braissant O., Astasov-Frauenhoffer M., Waltimo T., Bonkat G. (2020). A review of methods to determine viability, vitality, and metabolic rates in microbiology. Front. Microbiol..

[35] Tennant R.W., Margolin B.H., Shelby M.D., Zeiger E., Haseman J.K., Spalding J., Caspary W., Resnick M., Stasiewicz S., Anderson B. (1987). Prediction of chemical carcinogenicity in rodents from in vitro genetic toxicity assays. Science.

[36] Parry J.M., Parry E.M. (2012).

[37] Gatehouse D. (2012).

