# Supplementary material for: Assessing the mutagenic potential of methyl phenlactonoate 3 and Nijmegen-1 in bacterial reverse mutation assays
Source: Heliyon. 2024 Nov 20;10(23):e40526. doi: 10.1016/j.heliyon.2024.e40526 (PMC11625272; doi:10.1016/j.heliyon.2024.e40526)
Supplement: Multimedia component 3 [file mmc3.pptx]

## Slide 1
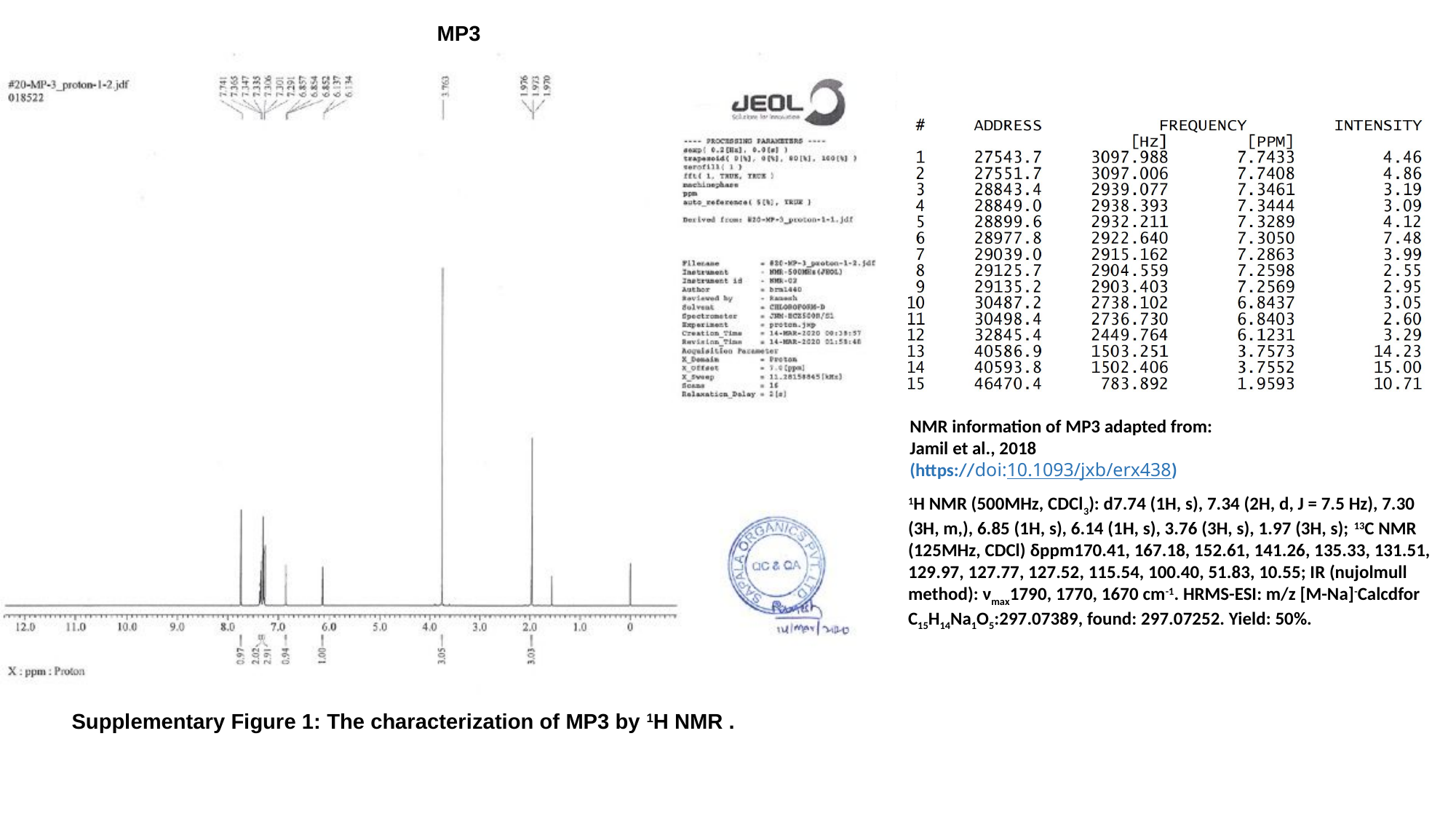

MP3
NMR information of MP3 adapted from:
Jamil et al., 2018
(https://doi:10.1093/jxb/erx438)
1H NMR (500MHz, CDCl3): d7.74 (1H, s), 7.34 (2H, d, J = 7.5 Hz), 7.30 (3H, m,), 6.85 (1H, s), 6.14 (1H, s), 3.76 (3H, s), 1.97 (3H, s); 13C NMR (125MHz, CDCl) δppm170.41, 167.18, 152.61, 141.26, 135.33, 131.51, 129.97, 127.77, 127.52, 115.54, 100.40, 51.83, 10.55; IR (nujolmull method): νmax1790, 1770, 1670 cm-1. HRMS-ESI: m/z [M-Na]-Calcdfor C15H14Na1O5:297.07389, found: 297.07252. Yield: 50%.
Supplementary Figure 1: The characterization of MP3 by 1H NMR .

## Slide 2
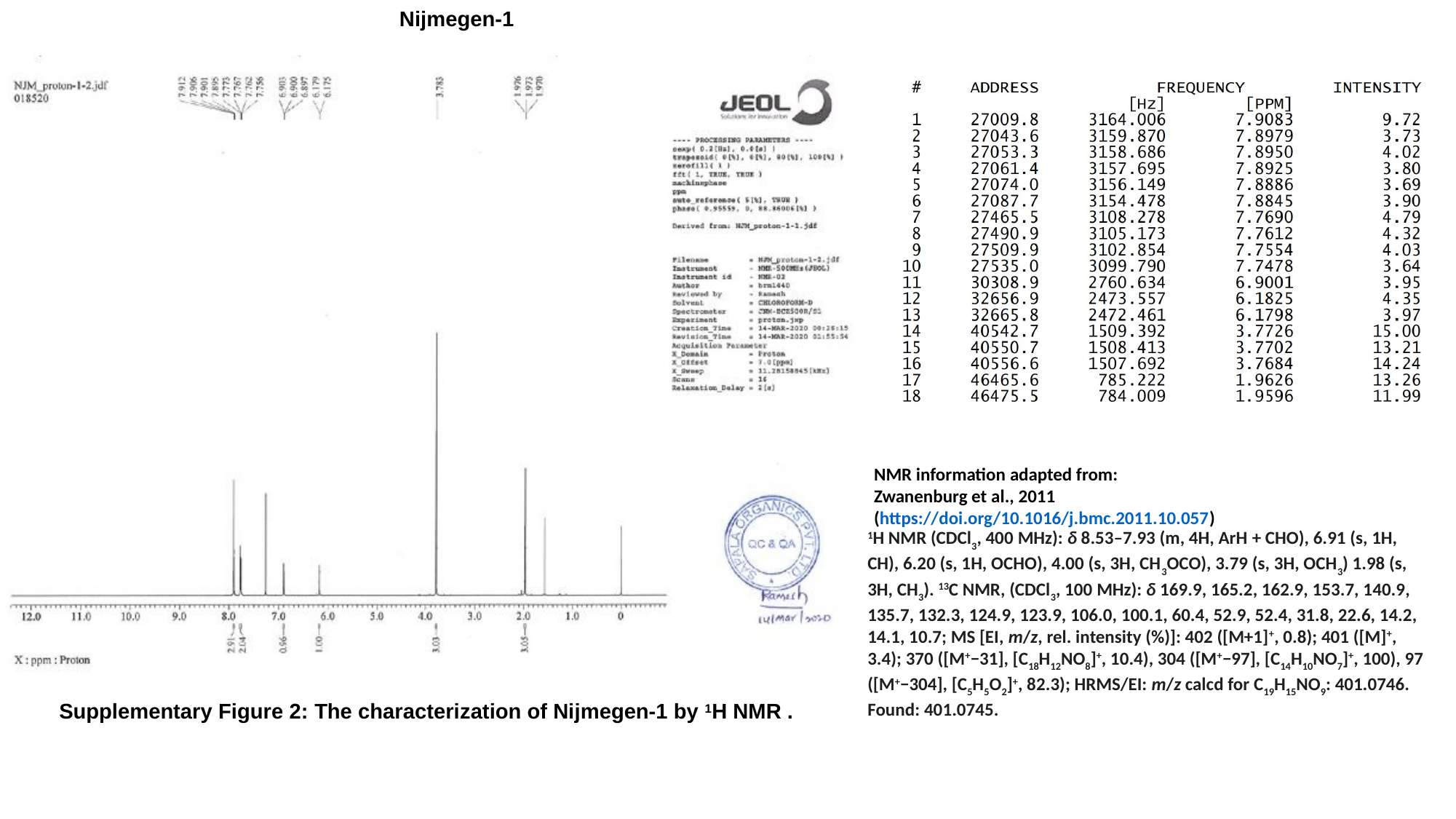

Nijmegen-1
NMR information adapted from:
Zwanenburg et al., 2011
(https://doi.org/10.1016/j.bmc.2011.10.057)
1H NMR (CDCl3, 400 MHz): δ 8.53–7.93 (m, 4H, ArH + CHO), 6.91 (s, 1H, CH), 6.20 (s, 1H, OCHO), 4.00 (s, 3H, CH3OCO), 3.79 (s, 3H, OCH3) 1.98 (s, 3H, CH3). 13C NMR, (CDCl3, 100 MHz): δ 169.9, 165.2, 162.9, 153.7, 140.9, 135.7, 132.3, 124.9, 123.9, 106.0, 100.1, 60.4, 52.9, 52.4, 31.8, 22.6, 14.2, 14.1, 10.7; MS [EI, m/z, rel. intensity (%)]: 402 ([M+1]+, 0.8); 401 ([M]+, 3.4); 370 ([M+−31], [C18H12NO8]+, 10.4), 304 ([M+−97], [C14H10NO7]+, 100), 97 ([M+−304], [C5H5O2]+, 82.3); HRMS/EI: m/z calcd for C19H15NO9: 401.0746. Found: 401.0745.
Supplementary Figure 2: The characterization of Nijmegen-1 by 1H NMR .

## Slide 3
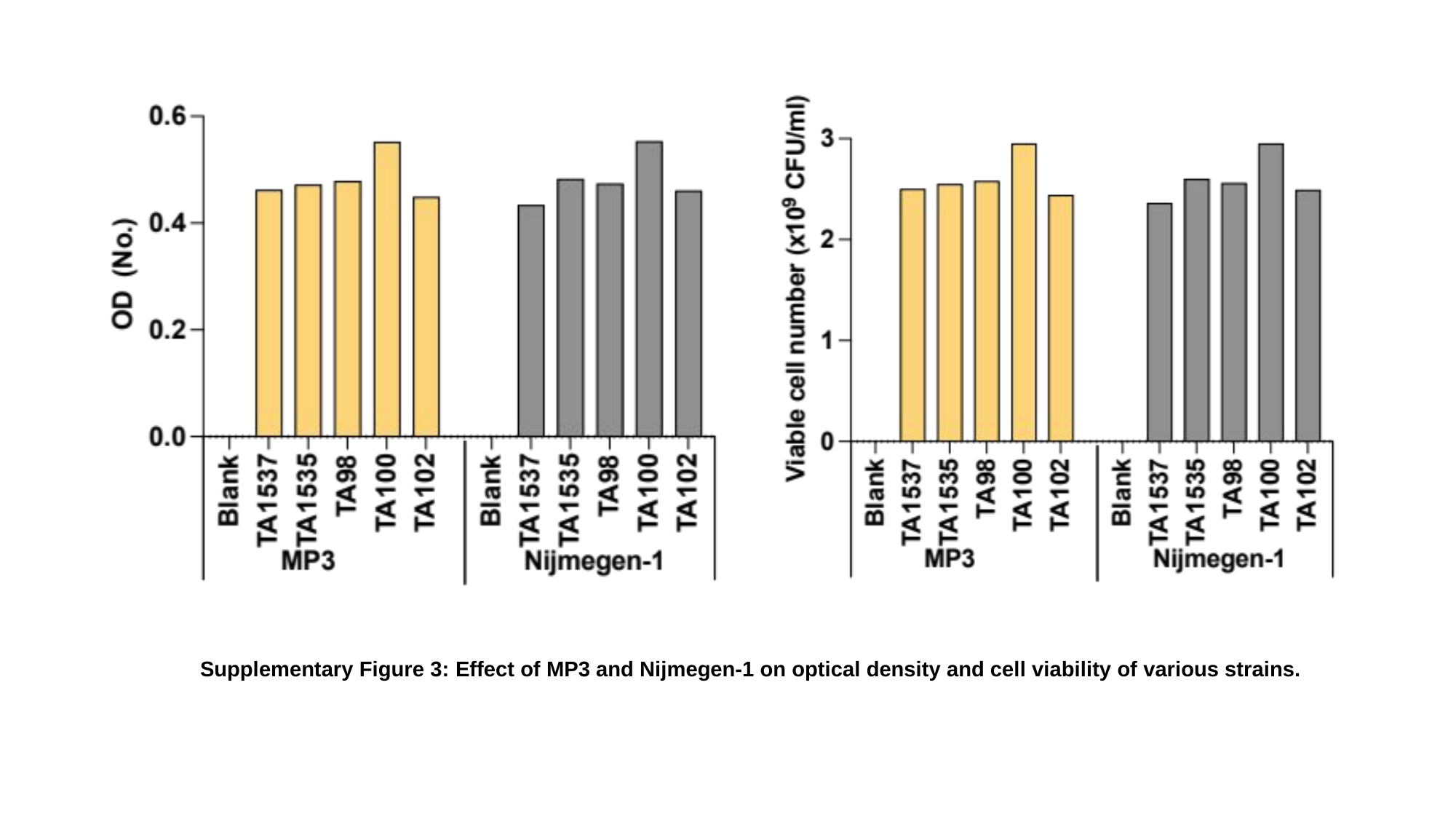

Supplementary Figure 3: Effect of MP3 and Nijmegen-1 on optical density and cell viability of various strains.

## Slide 4
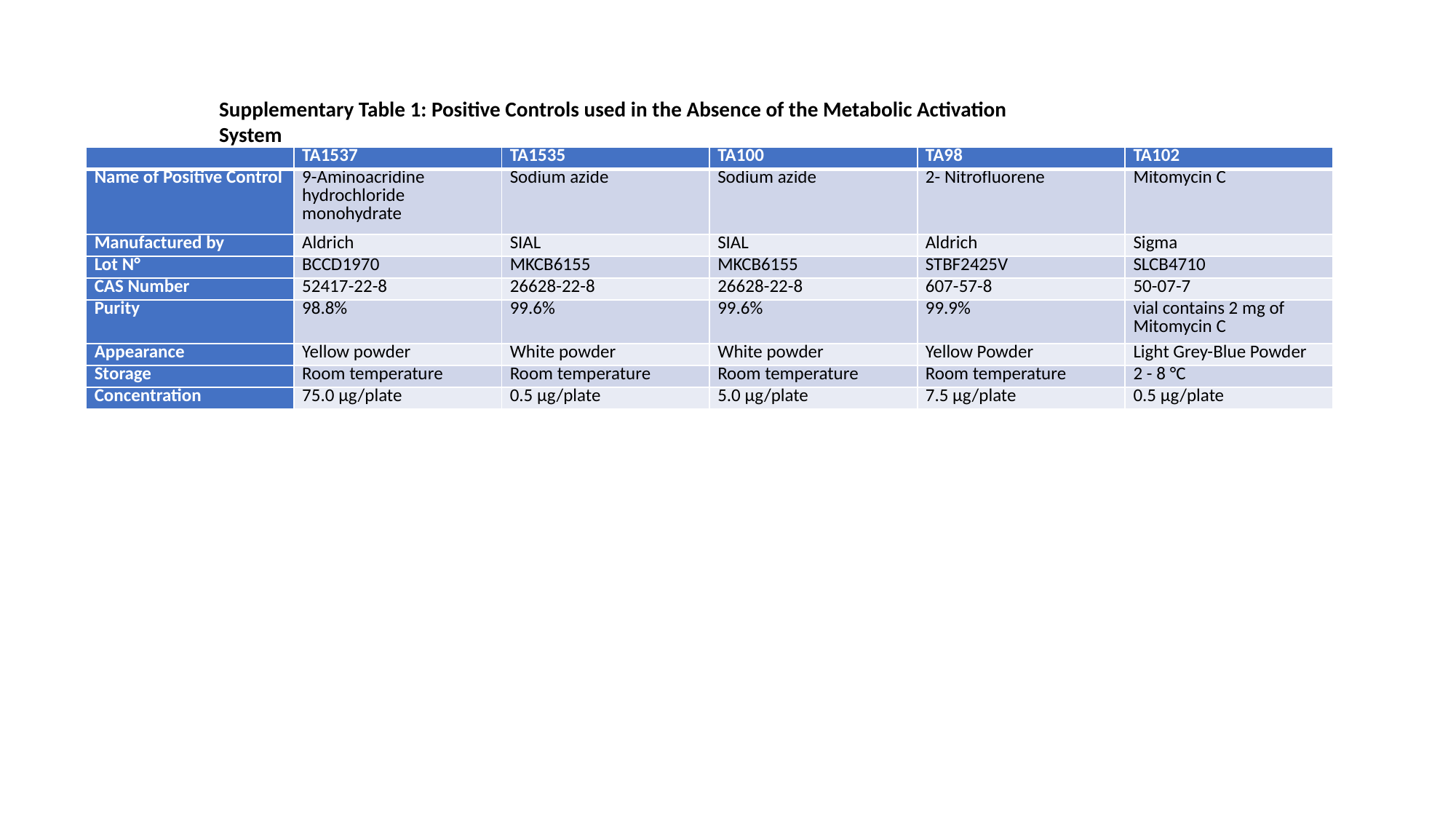

Supplementary Table 1: Positive Controls used in the Absence of the Metabolic Activation System
| | TA1537 | TA1535 | TA100 | TA98 | TA102 |
| --- | --- | --- | --- | --- | --- |
| Name of Positive Control | 9-Aminoacridine hydrochloride monohydrate | Sodium azide | Sodium azide | 2- Nitrofluorene | Mitomycin C |
| Manufactured by | Aldrich | SIAL | SIAL | Aldrich | Sigma |
| Lot N° | BCCD1970 | MKCB6155 | MKCB6155 | STBF2425V | SLCB4710 |
| CAS Number | 52417-22-8 | 26628-22-8 | 26628-22-8 | 607-57-8 | 50-07-7 |
| Purity | 98.8% | 99.6% | 99.6% | 99.9% | vial contains 2 mg of Mitomycin C |
| Appearance | Yellow powder | White powder | White powder | Yellow Powder | Light Grey-Blue Powder |
| Storage | Room temperature | Room temperature | Room temperature | Room temperature | 2 - 8 °C |
| Concentration | 75.0 μg/plate | 0.5 μg/plate | 5.0 μg/plate | 7.5 μg/plate | 0.5 μg/plate |

## Slide 5
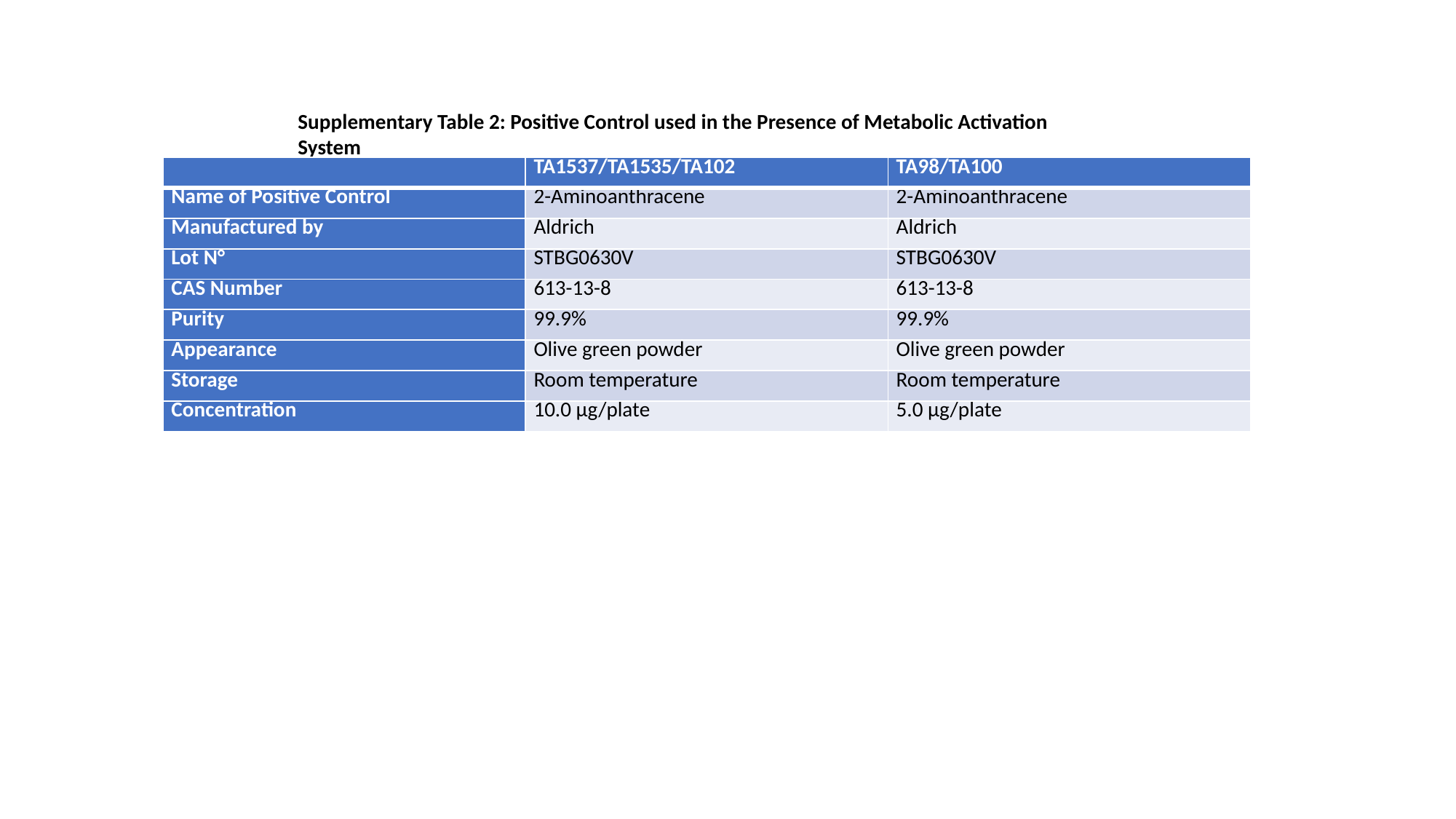

Supplementary Table 2: Positive Control used in the Presence of Metabolic Activation System
| | TA1537/TA1535/TA102 | TA98/TA100 |
| --- | --- | --- |
| Name of Positive Control | 2-Aminoanthracene | 2-Aminoanthracene |
| Manufactured by | Aldrich | Aldrich |
| Lot N° | STBG0630V | STBG0630V |
| CAS Number | 613-13-8 | 613-13-8 |
| Purity | 99.9% | 99.9% |
| Appearance | Olive green powder | Olive green powder |
| Storage | Room temperature | Room temperature |
| Concentration | 10.0 μg/plate | 5.0 μg/plate |

## Slide 6
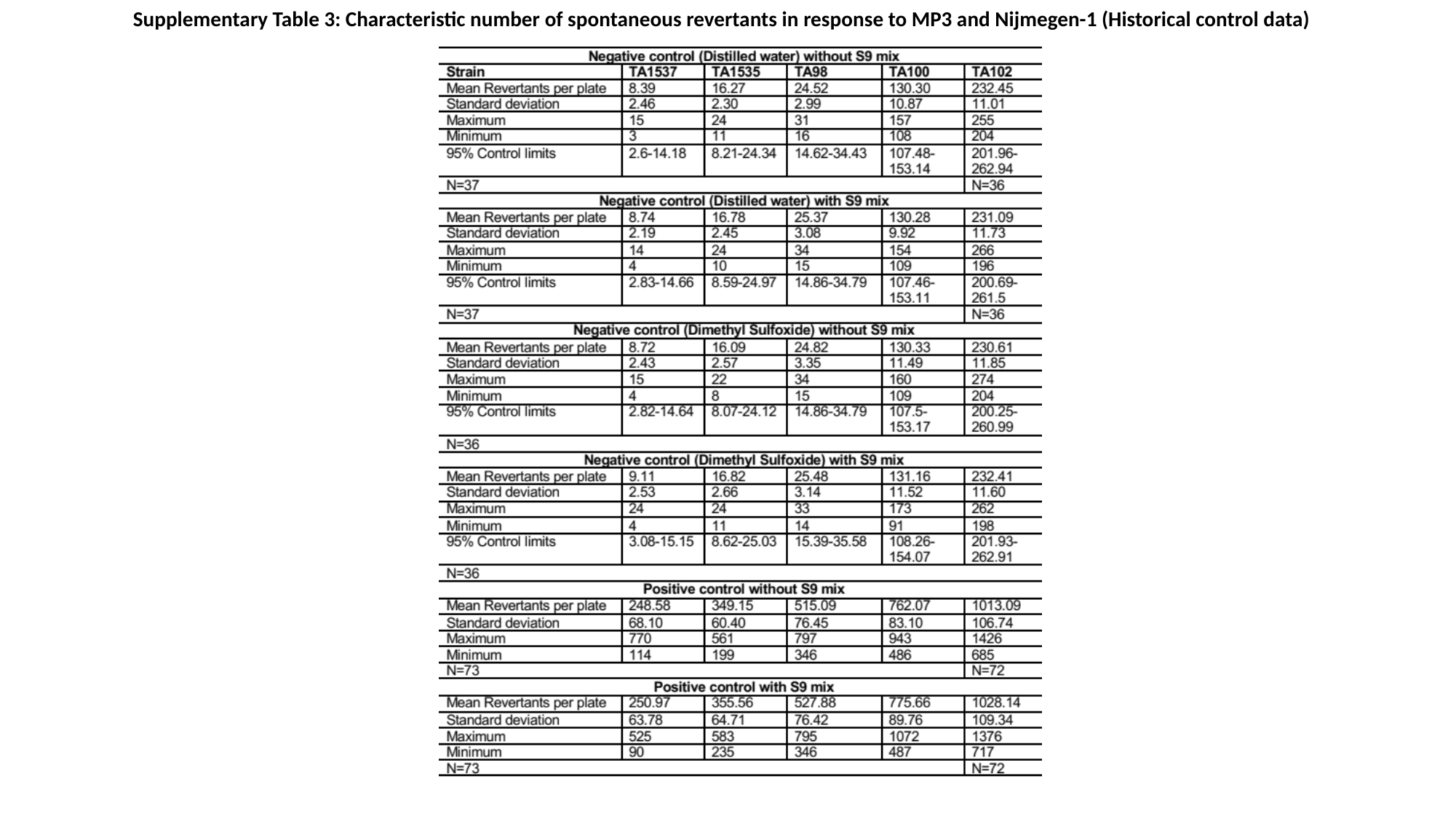

Supplementary Table 3: Characteristic number of spontaneous revertants in response to MP3 and Nijmegen-1 (Historical control data)

## Slide 7
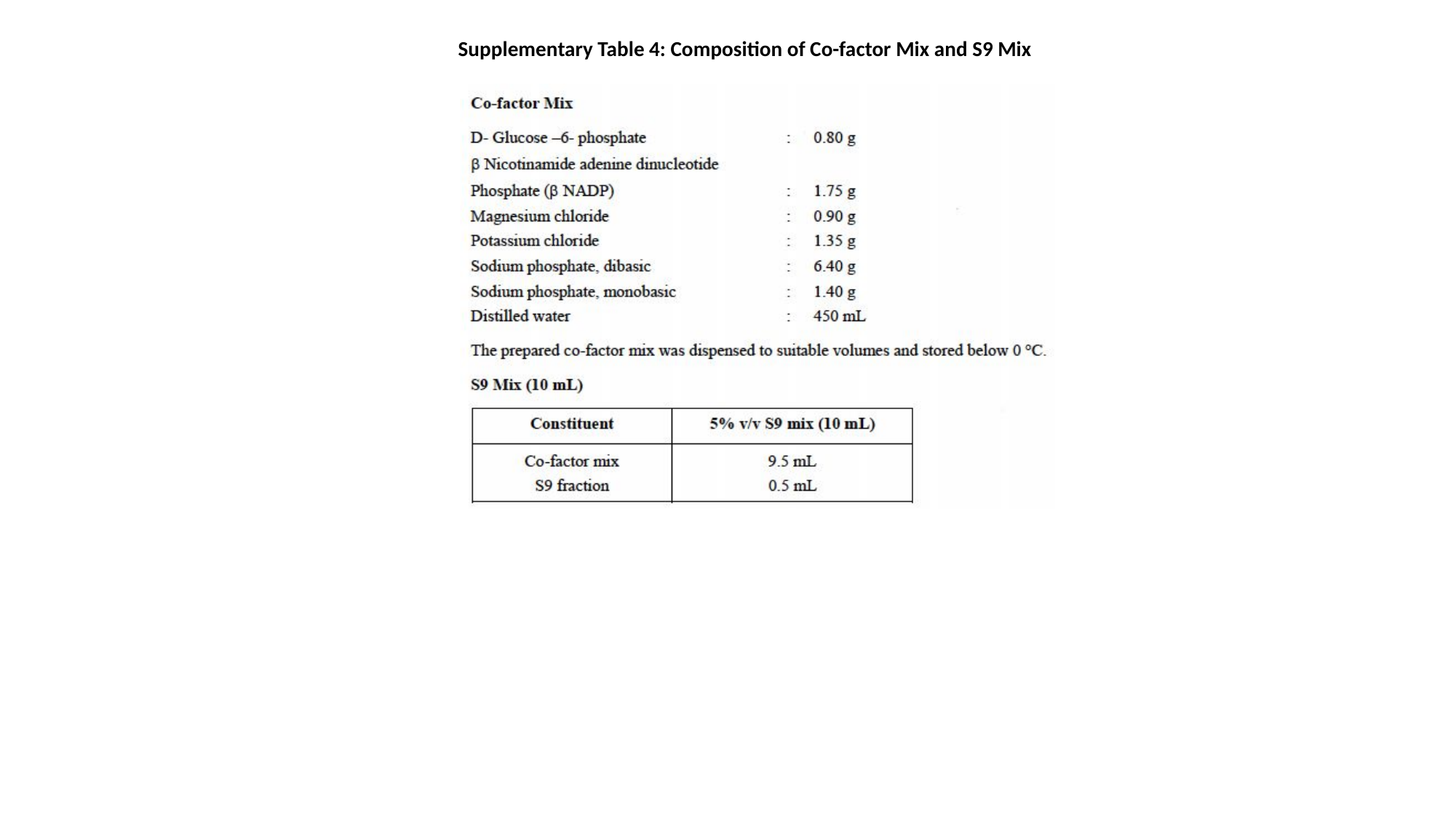

Supplementary Table 4: Composition of Co-factor Mix and S9 Mix

## Slide 8
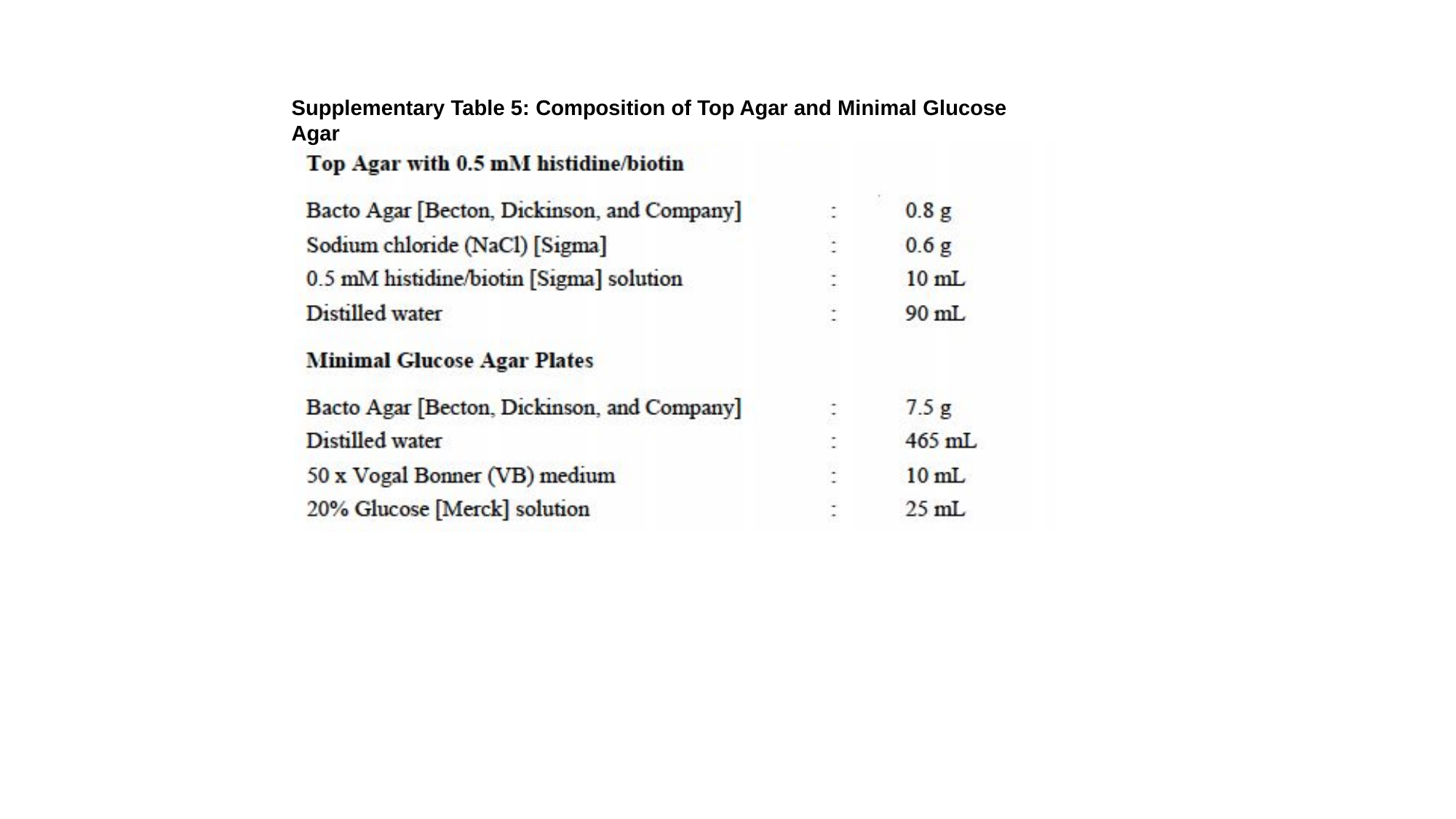

Supplementary Table 5: Composition of Top Agar and Minimal Glucose Agar

## Slide 9
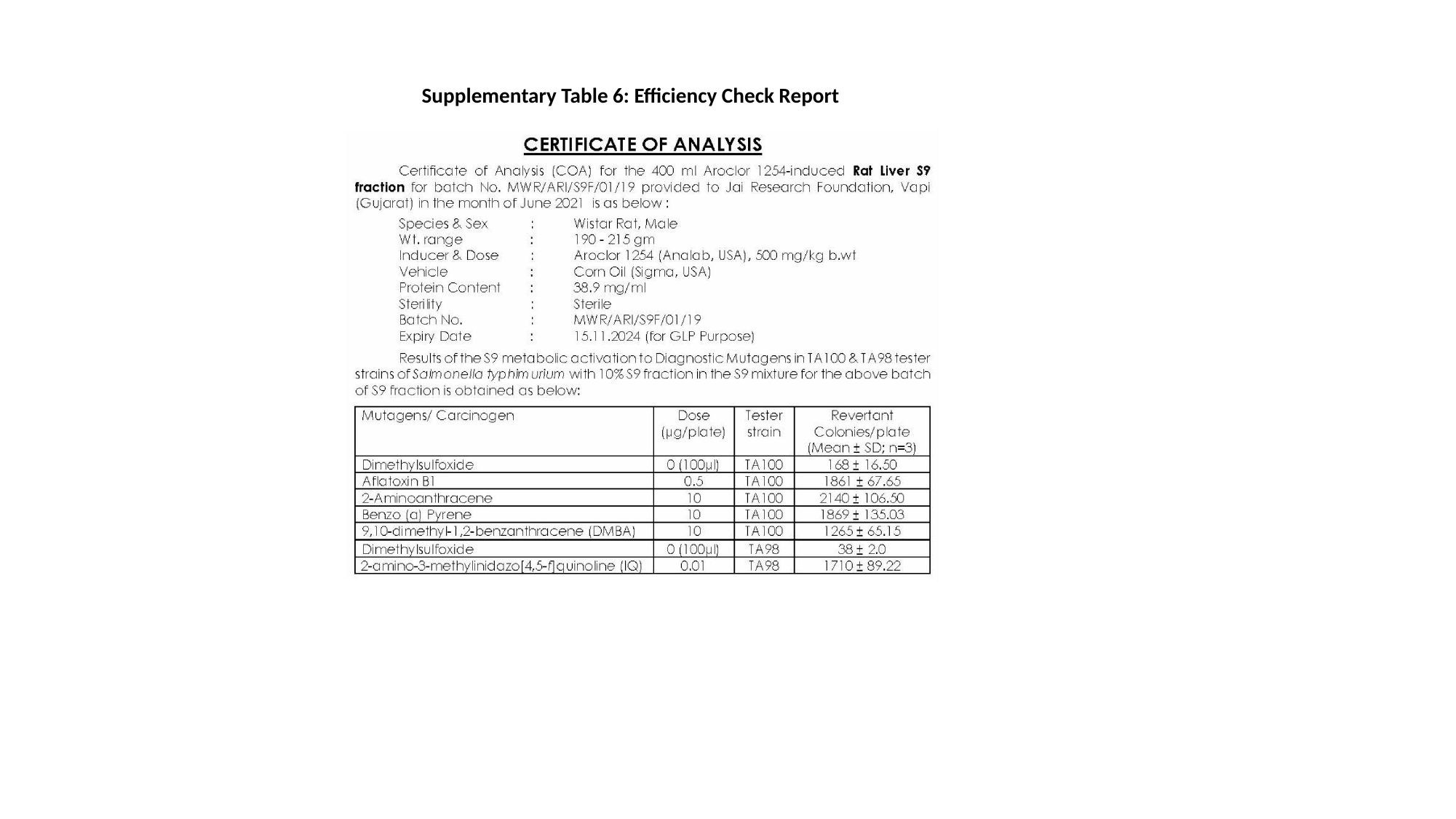

Supplementary Table 6: Efficiency Check Report

## Slide 10
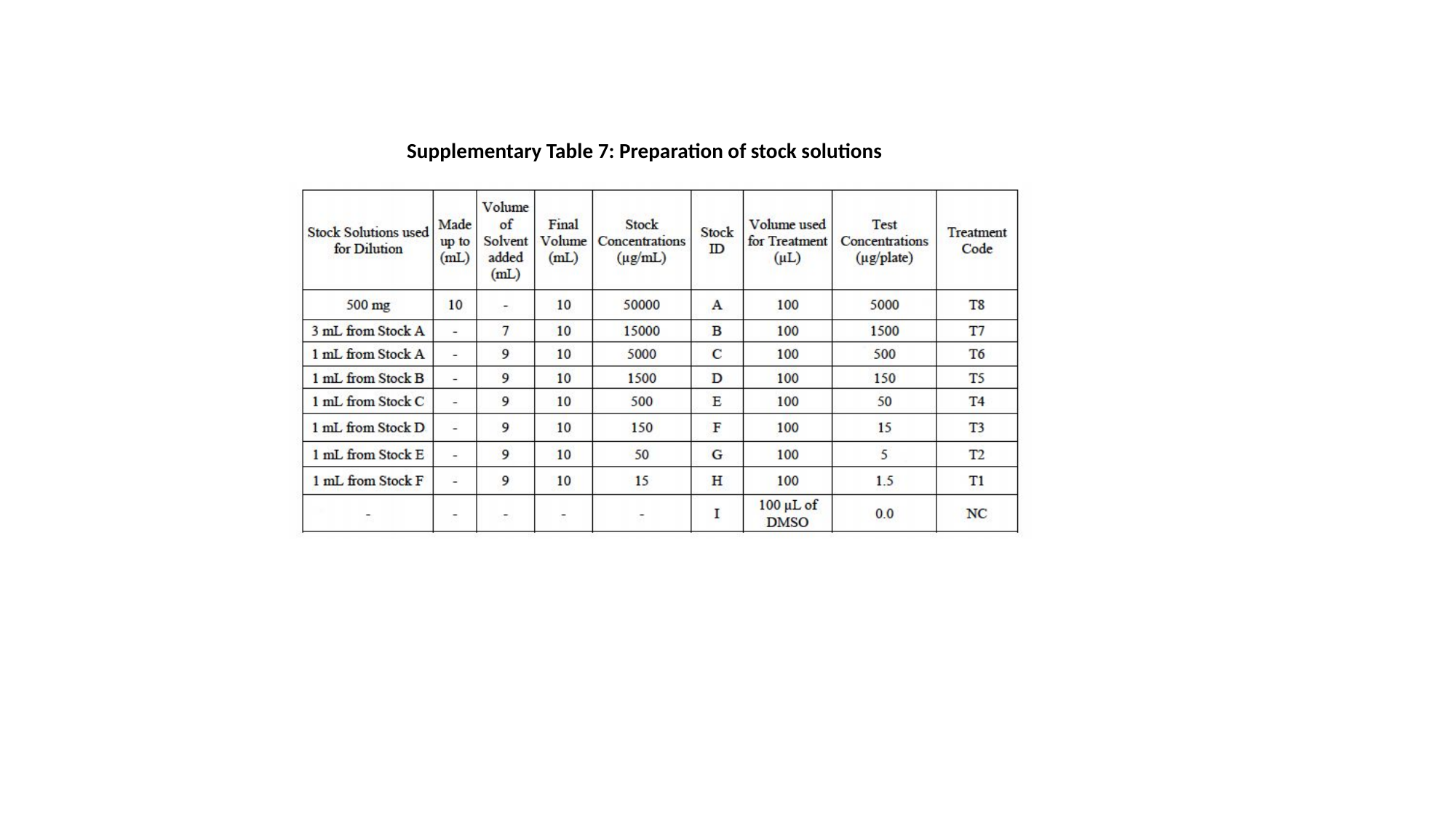

Supplementary Table 7: Preparation of stock solutions

## Slide 11
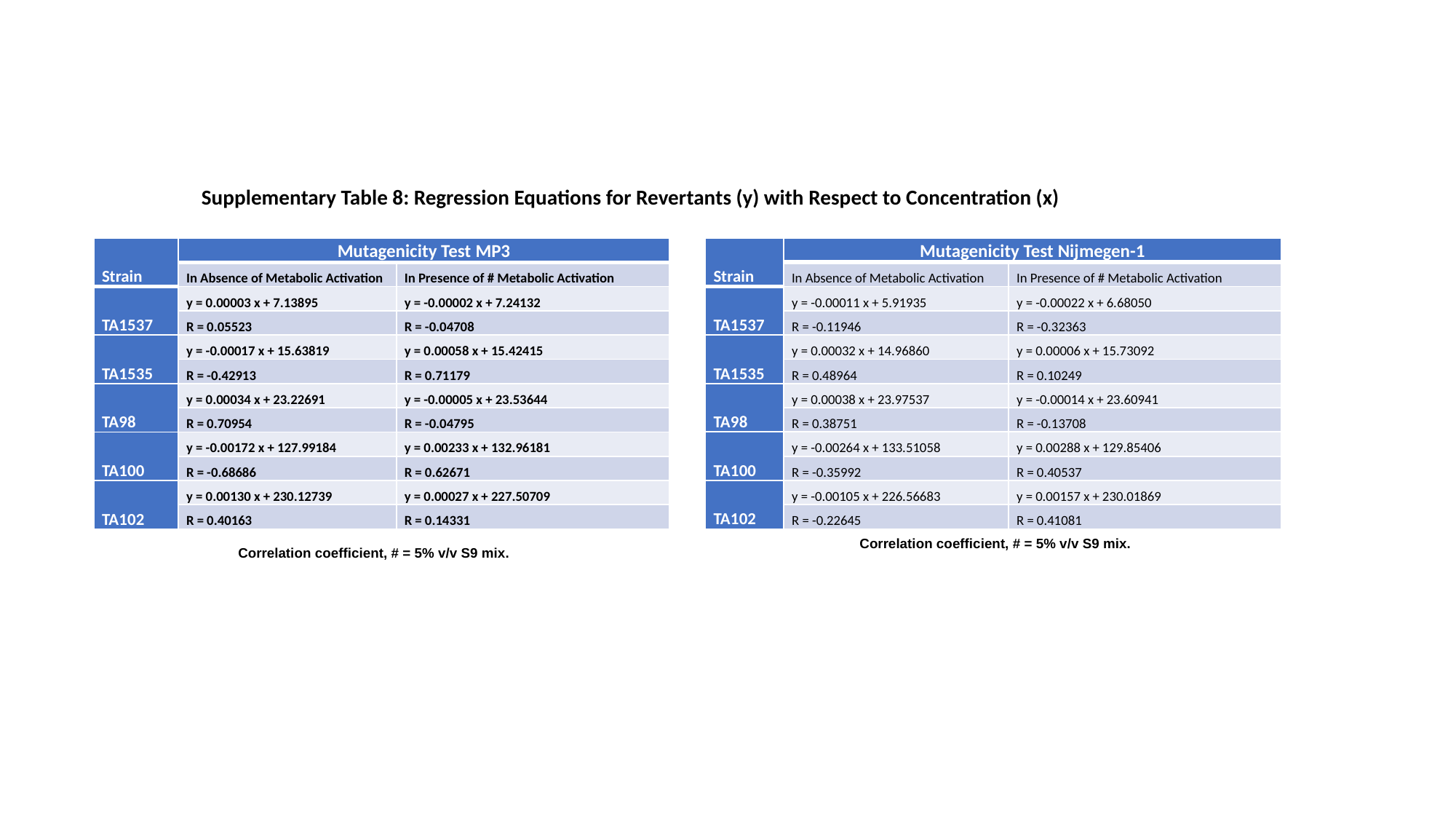

Supplementary Table 8: Regression Equations for Revertants (y) with Respect to Concentration (x)
| Strain | Mutagenicity Test Nijmegen-1 | |
| --- | --- | --- |
| | In Absence of Metabolic Activation | In Presence of # Metabolic Activation |
| TA1537 | y = -0.00011 x + 5.91935 | y = -0.00022 x + 6.68050 |
| | R = -0.11946 | R = -0.32363 |
| TA1535 | y = 0.00032 x + 14.96860 | y = 0.00006 x + 15.73092 |
| | R = 0.48964 | R = 0.10249 |
| TA98 | y = 0.00038 x + 23.97537 | y = -0.00014 x + 23.60941 |
| | R = 0.38751 | R = -0.13708 |
| TA100 | y = -0.00264 x + 133.51058 | y = 0.00288 x + 129.85406 |
| | R = -0.35992 | R = 0.40537 |
| TA102 | y = -0.00105 x + 226.56683 | y = 0.00157 x + 230.01869 |
| | R = -0.22645 | R = 0.41081 |
| Strain | Mutagenicity Test MP3 | |
| --- | --- | --- |
| | In Absence of Metabolic Activation | In Presence of # Metabolic Activation |
| TA1537 | y = 0.00003 x + 7.13895 | y = -0.00002 x + 7.24132 |
| | R = 0.05523 | R = -0.04708 |
| TA1535 | y = -0.00017 x + 15.63819 | y = 0.00058 x + 15.42415 |
| | R = -0.42913 | R = 0.71179 |
| TA98 | y = 0.00034 x + 23.22691 | y = -0.00005 x + 23.53644 |
| | R = 0.70954 | R = -0.04795 |
| TA100 | y = -0.00172 x + 127.99184 | y = 0.00233 x + 132.96181 |
| | R = -0.68686 | R = 0.62671 |
| TA102 | y = 0.00130 x + 230.12739 | y = 0.00027 x + 227.50709 |
| | R = 0.40163 | R = 0.14331 |
Correlation coefficient, # = 5% v/v S9 mix.
Correlation coefficient, # = 5% v/v S9 mix.
